# Supplementary material for: Mutant huntingtin protein decreases with CAG repeat expansion: implications for therapeutics and bioassays
Source: Brain Commun. 2024 Nov 15;6(6):fcae410. doi: 10.1093/braincomms/fcae410 (PMC11660907; doi:10.1093/braincomms/fcae410)
Supplement: fcae410_Supplementary_Data [file fcae410_supplementary_data.pdf]

Mutant HTT protein decreases with CAG repeat expansion: implications for therapeutics and bioassays.

Christian Landles, Georgina F. Osborne, Jemima Phillips, Maria Canibano Pico,

Iulia M. Nita, Nadira Ali, Konstantin Bobkov, Jonathan R. Greene,

Kirupa Sathasivam and Gillian P. Bates

Supplementary Table 1. CAG repeat sizes of the mice used in this study.

Knock-in line	Heterozygotes	Homozygotes<br>(small allele)<br>(large allele)
*Hdh*Q20	18	18<br>18
*Hdh*Q50	50.1 ± 0.24	49.4 ± 0.78<br>49.4 ± 0.78
*Hdh*Q80	80.1 ± 0.67	80.9 ± 1.51<br>81.4 ± 1.42
*Hdh*Q111	114.7 ± 2.38	110.4 ± 3.5<br>114.0 ± 2.74
CAG140	144.9 ± 2.62	140.5 ± 4.85<br>146.8 ± 2.10
zQ175	190.3 ± 5.98	184.1 ± 5.24<br>194.8 ± 3.96

Supplementary Table 2. Summary of Antibodies.

Name	Immunogen	Epitope	Species	Reference / Source
2B7	Human HTT peptide: aa 1-17[1]	LMKAFE*	Mouse Monoclonal	CHDI Foundation
MW1	Human HTT Exon1 (67Q)[2]	PolyQ	Mouse Monoclonal	CHDI Foundation
4C9	Human HTT peptide: aa 51-71[3]		Mouse Monoclonal	CHDI Foundation
MW8	AEEPLHRPK (67Q)[2]	Within: AEEPLHRP[4] Ends at proline	Mouse Monoclonal	CHDI Foundation
MAB5490	Human HTT: aa 115-129[5]	Within: QSVRNSPEFQKLLGI (mouse L)	Mouse Monoclonal	Sigma-Aldrich, MAB5490
MAB2166	HTT fusion protein: aa 181-810[6]	Within aa 443-457 GKVLLGEEEALEDDS [7]	Mouse Monoclonal	Sigma-Aldrich, MAB2166
D7F7		Within: aa 1214-1223* QSDTSGPVTT (mouse A)	Rabbit Monoclonal	Cell Signaling Technology #5656
HDAC4 (DM15)	Peptide: human HDAC4		Rabbit polyclonal	Merck Life Sciences pAb#H9536

*Information provided by the CHDI Foundation

Supplementary Table 2 References

1. Weiss A, Abramowski D, Bibel M, et al. Single-step detection of mutant huntingtin in animal and human tissues: A bioassay for Huntington's disease. *Analytical biochemistry*. Dec 1 2009;395(1):8-15. doi:S0003-2697(09)00541-7 [pii]
10.1016/j.ab.2009.08.001
2 .Ko J, Ou S, Patterson PH. New anti-huntingtin monoclonal antibodies: implications for huntingtin conformation and its binding proteins. *Brain research bulletin*. Oct-Nov 1 2001;56(3-4):319-329.
3. Baldo B, Paganetti P, Grueninger S, et al. TR-FRET-based duplex immunoassay reveals an inverse correlation of soluble and aggregated mutant huntingtin in huntington's disease. *Chemistry & biology*. Feb 24 2012;19(2):264-275. doi:S1074-5521(12)00016-6 [pii]
10.1016/j.chembiol.2011.12.020
4. Landles C, Sathasivam K, Weiss A, et al. Proteolysis of mutant huntingtin produces an exon 1 fragment that accumulates as an aggregated protein in neuronal nuclei in Huntington disease. *J Biol Chem*. Mar 19 2010;285(12):8808-8823. doi:M109.075028 [pii]
10.1074/jbc.M109.075028
5. Lunkes A, Lindenberg KS, Ben-Haiem L, et al. Proteases acting on mutant huntingtin generate cleaved products that differentially build up cytoplasmic and nuclear inclusions. *Molecular cell*. Aug 2002;10(2):259-269.
6. Trottier Y, Devys D, Imbert G, et al. Cellular localization of the Huntington's disease protein and discrimination of the normal and mutated form [see comments]. *Nature genetics*. 1995;10(1):104-110.
7. Cong SY, Pepers BA, Roos RA, Van Ommen GJ, Dorsman JC. Epitope mapping of monoclonal antibody 4C8 recognizing the protein huntingtin. *Hybridoma (Larchmt)*. Oct 2005;24(5):231-5. doi:10.1089/hyb.2005.24.231

Supplementary Table 3. Antibody and lysate concentrations for HTRF assays.

Figure Number	Antibody Pairing	Donor (ng / well)	Acceptor (ng / well)	Lysate Dilution Concentration
Figure 3	MW1-Tb : MAB5490-d2	1 ng	20 ng	5%
	MW1-Tb : MAB2166-d2	1 ng	20 ng	5%
	MW1-Tb : D7F7-d2	1 ng	20 ng	5%
	MAB5490-Tb : MW1-d2	1 ng	20 ng	5%
	MAB2166-Tb : MW1-d2	1 ng	20 ng	5%
	D7F7-Tb : MW1-d2	1 ng	20 ng	5%
Figure 4	4C9-Tb : MAB5490-d2	1 ng	20 ng	10%
	4C9-Tb : MAB2166-d2	1 ng	20 ng	10%
	4C9-Tb : D7F7-d2	1 ng	20 ng	10%
	MAB5490-Tb : 4C9-488	1 ng	20 ng	10%
	MAB2166-Tb : 4C9-488	1 ng	20 ng	10%
	D7F7-Tb : 4C9-488	1 ng	20 ng	10%
Figure 5	2B7-Tb : MW8-d2	1 ng	40 ng	10%
	MW1-Tb : MW8-d2	1 ng	40 ng	10%
	4C9-Tb : MW8-d2	1 ng	20 ng	10% Crude Lysate
Figure 6	2B7-Tb : MW1-d2	1 ng	20 ng	2.5%
	2B7-Tb : 4C9-488	1 ng	20 ng	5%
	MW1-Tb : 2B7-d2	1 ng	20 ng	2.5%
	MW1-Tb : 4C9-488	1 ng	20 ng	5%
	4C9-Tb : 2B7-d2	1 ng	20 ng	5%
	4C9-Tb : MW1-d2	1 ng	20 ng	5%
Figure 7	2B7-Tb : MAB5490-d2	1 ng	20 ng	10%
	2B7-Tb : MAB2166-d2	1 ng	20 ng	10%
	2B7-Tb : D7F7-d2	1 ng	20 ng	10%
	MAB5490-Tb : 2B7-d2	1 ng	20 ng	10%
	MAB2166-Tb : 2B7-d2	1 ng	20 ng	10%
	D7F7-Tb : 2B7-d2	1 ng	20 ng	10%
Figure 8	MAB5490-Tb : MAB2166-d2	1 ng	20 ng	5%
	MAB5490-Tb : D7F7-d2	1 ng	20 ng	5%
	MAB2166-Tb : MAB5490-d2	1 ng	20 ng	5%
	MAB2166-Tb : D7F7-d2	1 ng	20 ng	5%
	D7F7-Tb : MAB5490-d2	1 ng	20 ng	5%
	D7F7-Tb : MAB2166-d2	1 ng	20 ng	5%

NB: Final antibody working conditions and lysate dilutions must always be established by end user, since plate reader sensitivities will change depending on make/model.

Supplementary Table 4. Antibody and lysate concentrations for MSD assays.

Figure Number	Antibody Pairing	Capture	Sulfo-tag (μg/mL)	Lysate Dilution Concentration
Figure 3	MW1-Capture : MAB5490-ST	MSD default	2.5 μg/mL	12%
	MW1-Capture : MAB2166-ST	MSD default	2.5 μg/mL	12%
	MW1-Capture : D7F7-St	MSD default	2.5 μg/mL	12%
	MAB5490-Capture : MW1-ST	MSD default	1.5 μg/mL	12%
	MAB2166-Capture : MW1-ST	MSD default	1.5 μg/mL	12%
	D7F7-Capture : MW1-ST	MSD default	1.5 μg/mL	12%
Figure 4	4C9-Capture : MAB5490-ST	MSD default	2.5 μg/mL	12%
	4C9-Capture : MAB2166-ST	MSD default	2.5 μg/mL	12%
	4C9-Capture : D7F7-ST	MSD default	2.5 μg/mL	12%
	MAB5490-Capture : 4C9-ST	MSD default	2.5 μg/mL	12%
	MAB2166-Capture : 4C9-ST	MSD default	2.5 μg/mL	12%
	D7F7-Capture : 4C9-ST	MSD default	2.5 μg/mL	12%
Figure 5	2B7-Capture : MW8-ST	MSD default	2.5 μg/mL	18%
	MW1-Capture : MW8-ST	MSD default	2.5 μg/mL	18%
	4C9-Capture : MW8-ST	MSD default	2.5 μg/mL	18% Crude Lysate
Figure 6	2B7-Capture : MW1-ST	MSD default	1.5 μg/mL	12%
	2B7-Capture : 4C9-ST	MSD default	2.5 μg/mL	12%
	MW1-Capture : 2B7-ST	MSD default	2.5 μg/mL	12%
	MW1-Capture : 4C9-ST	MSD default	2.5 μg/mL	12%
	4C9-Capture : 2B7-ST	MSD default	2.5 μg/mL	12%
	4C9-Capture : MW1-ST	MSD default	1.5 μg/mL	12%
Figure 7	2B7-Capture : MAB5490-ST	MSD default	2.5 μg/mL	12%
	2B7-Capture : MAB2166-ST	MSD default	2.5 μg/mL	12%
	2B7-Capture : D7F7-ST	MSD default	2.5 μg/mL	12%
	MAB5490-Capture : 2B7-ST	MSD default	2.5 μg/mL	12%
	MAB2166-Capture : 2B7-ST	MSD default	2.5 μg/mL	12%
	D7F7-Capture : 2B7-ST	MSD default	2.5 μg/mL	12%
Figure 8	MAB5490-Capture : MAB2166-ST	MSD default	2.5 μg/mL	12%
	MAB5490-Capture: D7F7-ST	MSD default	2.5 μg/mL	12%
	MAB2166-Capture : MAB5490-ST	MSD default	2.5 μg/mL	12%
	MAB2166-Capture : D7F7-ST	MSD default	2.5 μg/mL	12%
	D7F7-Capture : MAB5490-ST	MSD default	2.5 μg/mL	12%
	D7F7-Capture : MAB2166-ST	MSD default	2.5 μg/mL	12%

Supplementary Table 5. PCR primers and probes.

Assay	Forward primer	Reverse primer	Probe
FL HTT (3'UTR)	CCT GGT ATG TGG ATC AGA AGT C	CAG GTA TGC CTA CTG GGT AAA T	5' FAM -AGC TCT TGC CAG ATG GTT CTG AGC 3' BHQ1
HTT1a	TCCTCATCAGGCCTAAGAGC TGG	GAGACCTCCTAAAAGC ATTATGTCATC	5' FAM AGTGCAGGACA GCGTGA 3' BHQ1

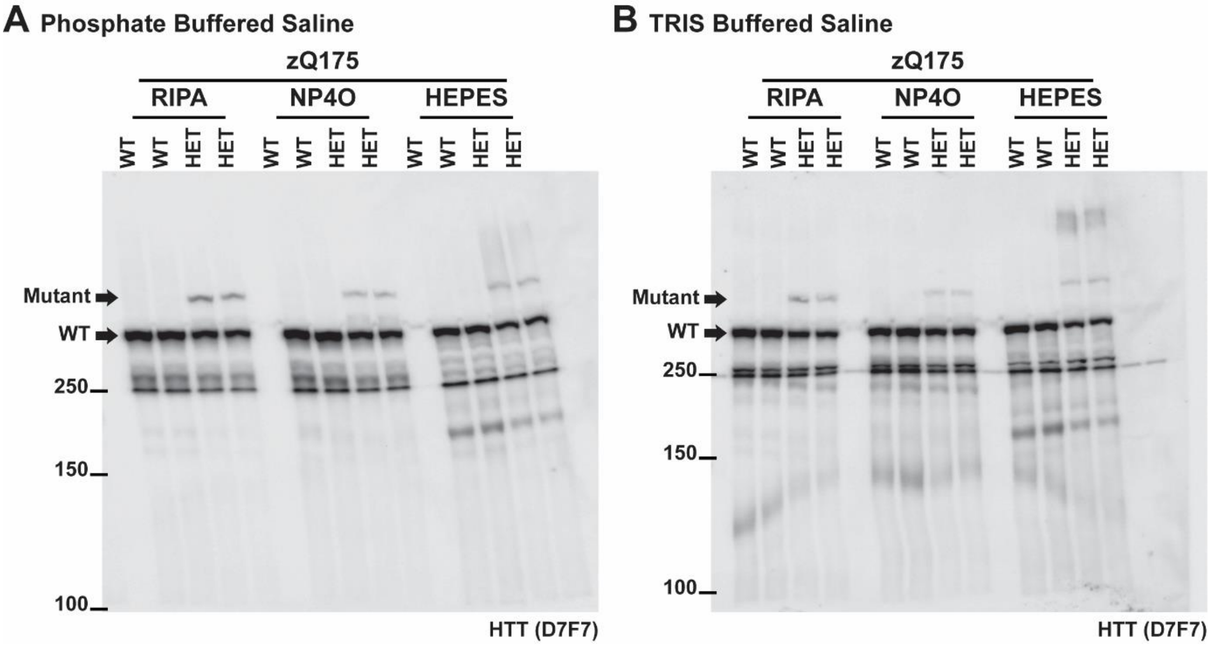

Supplementary Figure 1. Comparison of lysate buffers on the immunodetection of HTT with the D7F7 antibody on western blots of cortical lysates. Cortical lysates from wild-type and heterozygous zQ175 mice (n = 2 / genotype) prepared in RIPA, NP40 or HEPES buffers, separated by 7.5% SDS-PAGE, and immunoprobed with D7F7 in **(A)** PBST (phosphate buffered saline, 0.1% TWEEN 20) or **(B)** TBST (TRIS-buffered saline, 0.1% TWEEN 20). Blots immunoprobed in PBST were cleaner than those probed in TBST. The mutant HTT signal was marginally greater for lysates prepared in RIPA buffer and there was less non-specific background for the lysates prepared in RIPA buffer. WT = wild type, HET = heterozygote, size markers are in kDa.

Supplementary Figure 2

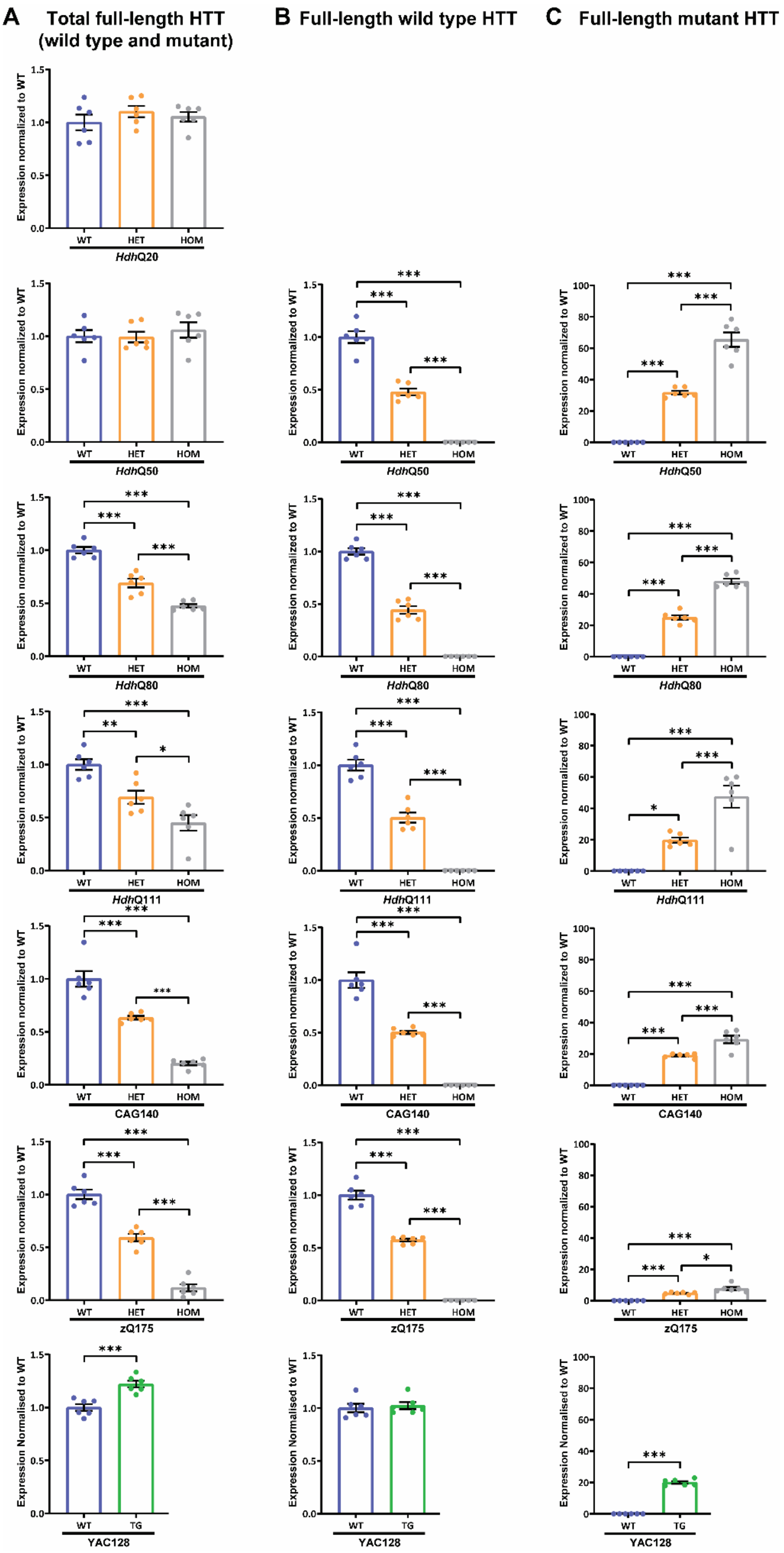

Supplementary Figure 2. Quantification of full-length wild-type and mutant HTT in cortical lysates in the allelic series of knock-in mouse lines and YAC128 mice. Quantification of the western blots in **Fig. 1 and Supplementary Figs 4–10**. **(A)** The levels of mutant and wild-type HTT were equivalent in the *Hdh*Q20 and *Hdh*Q50 lines. In contrast, the level of mutant HTT in the *Hdh*Q80, *Hdh*Q111, CAG140 and zQ175 cortical lysates decreased with increasing CAG repeat length. There was approximately 20% more HTT in YAC128 mice as compared to wild type. **(B)** Quantification of the wild-type HTT signals in the heterozygous lysates confirmed that these were approximately 50% of that in wild-type mice. Wild-type HTT was at endogenous levels in YAC128 mice. **(C)** Quantification of the mutant HTT signals in the homozygotes confirmed that these were approximately double that detected in the heterozygous lysates for all lines. Statistical analysis was by Student's *t*-test and one-way ANOVA with Bonferroni *post hoc* correction. Data points are individual mice. The test statistic, degrees of freedom and *p* values for the ANOVA are provided in **Supplementary Table 14**. Error bars are mean ± SEM. *$P \leq 0.05$, **$P \leq 0.01$, ***$P \leq 0.001$. WT = wild type, HET = heterozygote, HOM = homozygote, TG = transgenic.

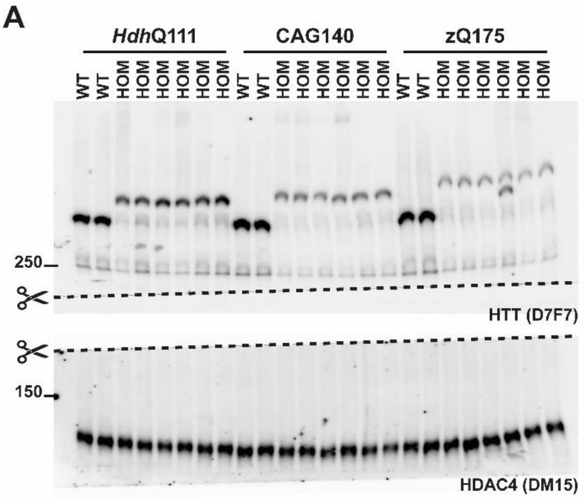

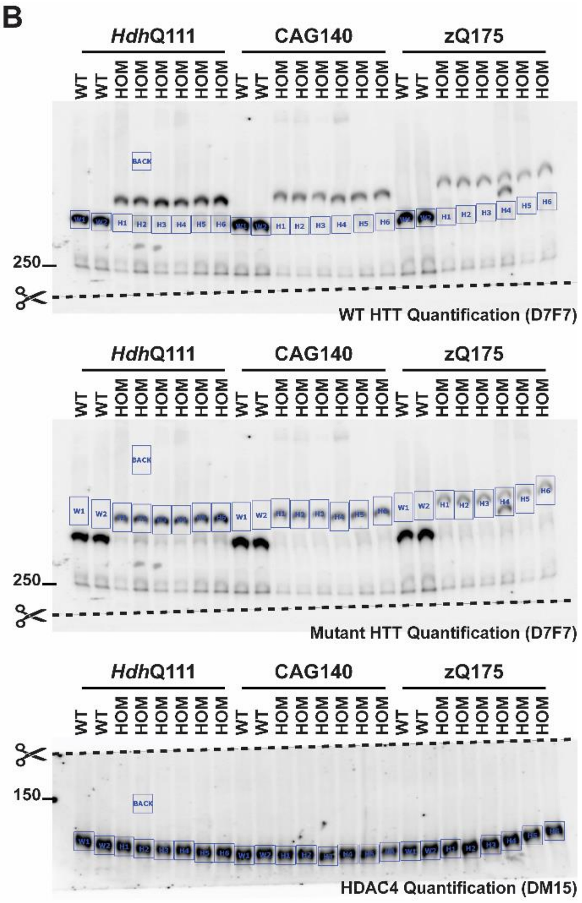

D

Mouse knock-in line	Total full-length HTT (WT or mutant), normalized to WT	
	WT	HOM
*Hdh*Q111	100	50
CAG140	100	23
zQ175	100	10

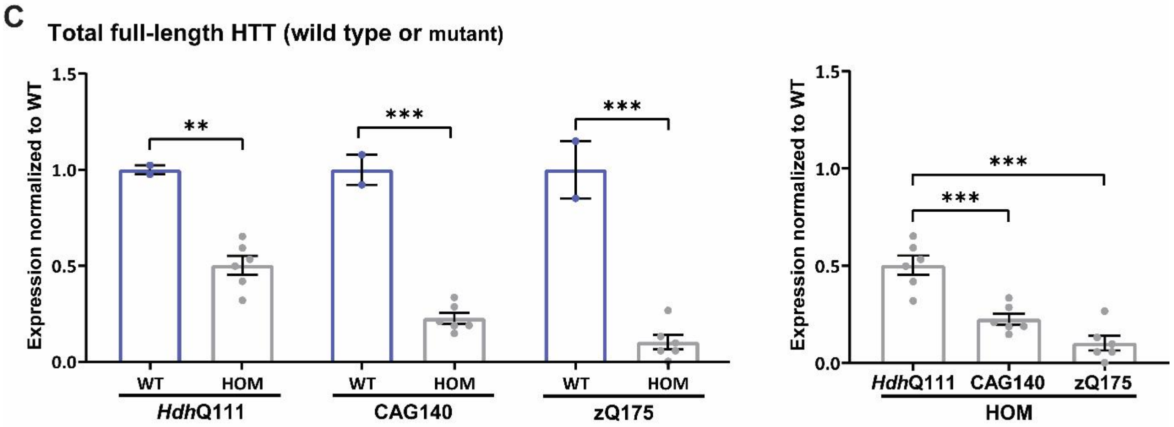

Supplementary Figure 3. Comparison of mutant HTT levels in the *Hdh*Q111, CAG140 and zQ175 cortical lysates. Uncropped blots for **Figure 1A.** **(A)** The western blot was cut where indicated. The top section probed with D7F7 for HTT, and the bottom section with DM15 for HDAC4 (n = 3/gender/genotype). **(B)** The signals were developed using a ChemiDoc-MP Imaging System (Bio-Rad) and quantified using Image Lab Software (Bio-Rad) as illustrated. **(C)** Mutant HTT levels in the homozygous lysates were normalized to wild-type HTT levels. **(D)** Summary of the level of full-length HTT in the knock-in mouse lines. Statistical analysis was by Student's *t*-test and one-way ANOVA with Bonferroni *post hoc* correction. The test statistic, degrees of freedom and *p* values for the ANOVA are provided in **Supplementary Table 15**. Error bars are mean ± SEM. $^{**}P \leq 0.01$, $^{***}P \leq 0.001$. WT = wild type, HOM = homozygote, BACK = background. Protein size markers are in kDa.

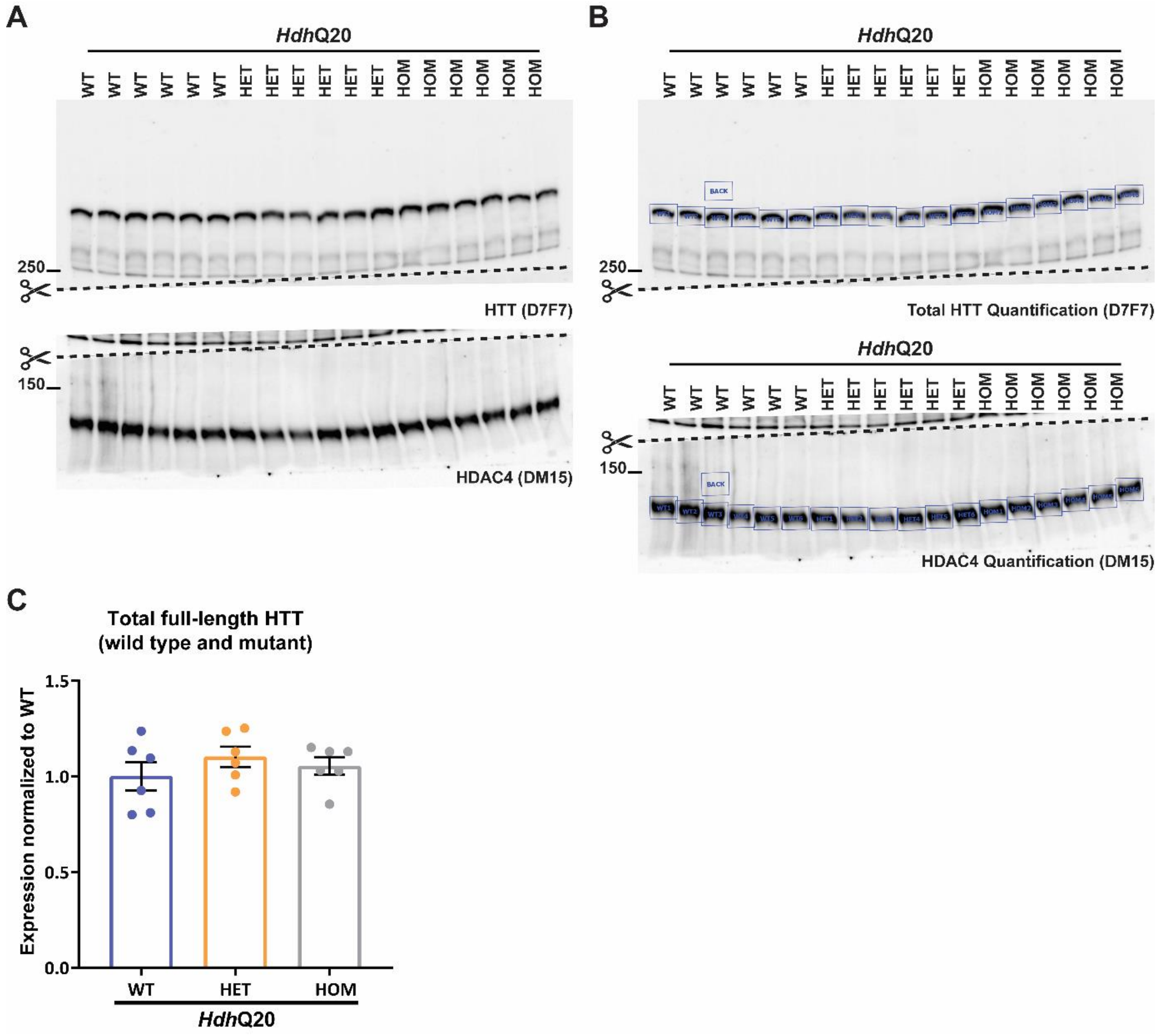

Supplementary Figure 4. Quantification of full-length HTT in cortical lysates from wild-type, heterozygous and homozygous mice from the *Hdh*Q20 colony. Uncropped blots for **Figure 1A. (A)** The western blot was cut where indicated. The top section was probed with D7F7 for HTT, and the bottom section with DM15 for HDAC4 (n = 3/gender/genotype). **(B)** The signals were developed using a ChemiDoc-MP Imaging System (Bio-Rad) and quantified using Image Lab Software (Bio-Rad) as illustrated. **(C)** Quantitation of total full-length HTT in the heterozygous and homozygous lysates normalized to wild-type levels. Statistical analysis was by one-way ANOVA with Bonferroni *post hoc* correction. The test statistic, degrees of freedom and *p* values for the ANOVA are provided in **Supplementary Table 16**. Error bars are mean ± SEM. WT = wild type, HET = heterozygote, HOM = homozygote, BACK = background. Protein size markers are in kDa.

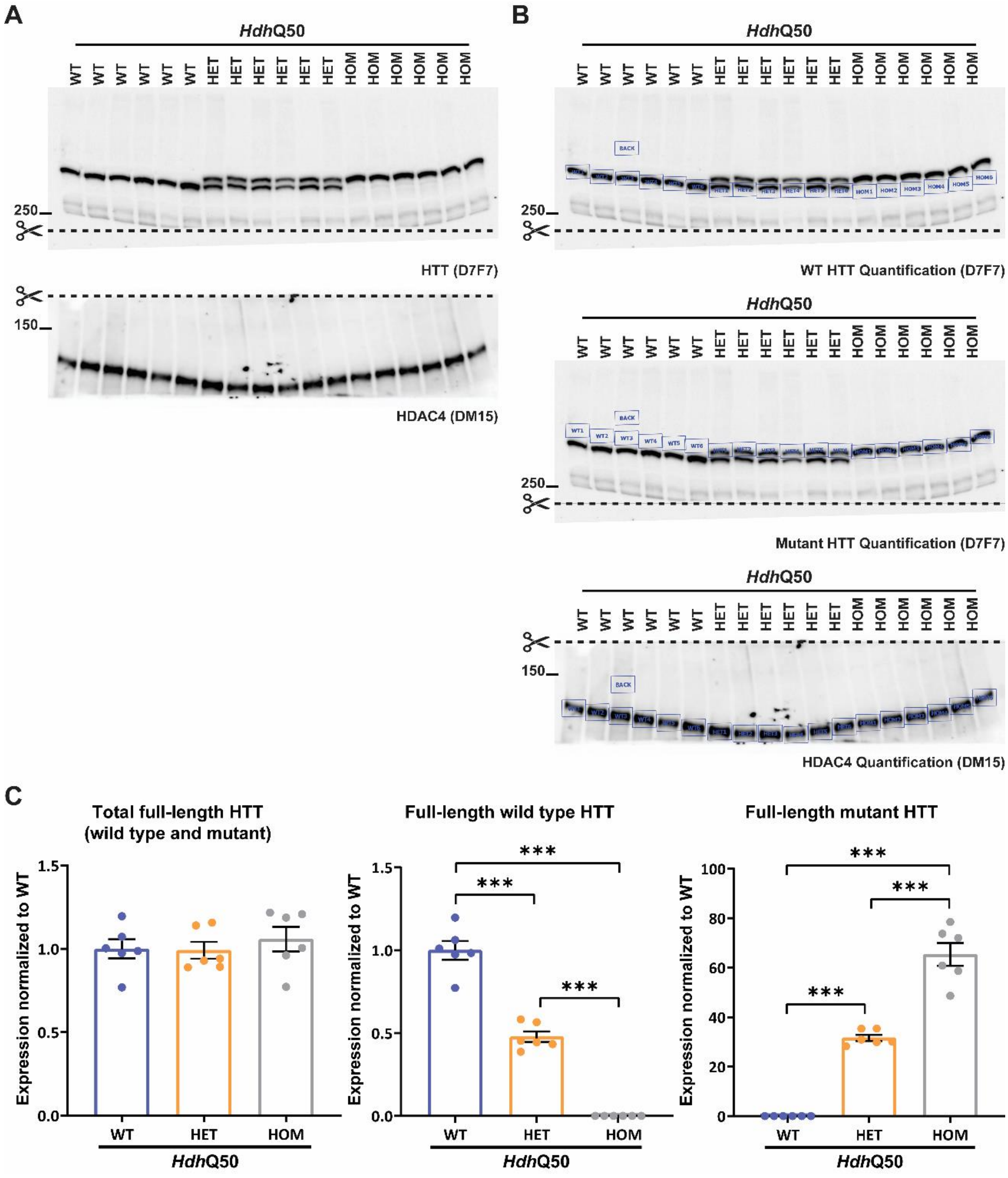

Supplementary Figure 5. Quantification of full-length HTT in cortical lysates from wild-type, heterozygous and homozygous mice from the *Hdh*Q50 colony. Uncropped blots for **Figure 1A. (A)** The western blot was cut where indicated. The top section probed with D7F7 for HTT, and the bottom section with DM15 for HDAC4 (n = 3/gender/genotype). **(B)** The signals were developed using a ChemiDoc-MP Imaging System (Bio-Rad) and quantified using Image Lab Software (Bio-Rad) as illustrated. **(C)** Total full-length HTT and full-length wild-type HTT in the heterozygous and homozygous lysates were normalized to wild-type HTT levels. Full-length mutant HTT levels in the heterozygous and homozygous lysates were normalized to wild-type background. Statistical analysis was by one-way ANOVA with Bonferroni *post hoc* correction. The test statistic, degrees of freedom and *p* values for the ANOVA are provided **in Supplementary Table 17**. Error bars are mean ± SEM. ***$P \leq 0.001$. WT = wild type, HET = heterozygote, HOM = homozygote, BACK = background. Protein size markers are in kDa.

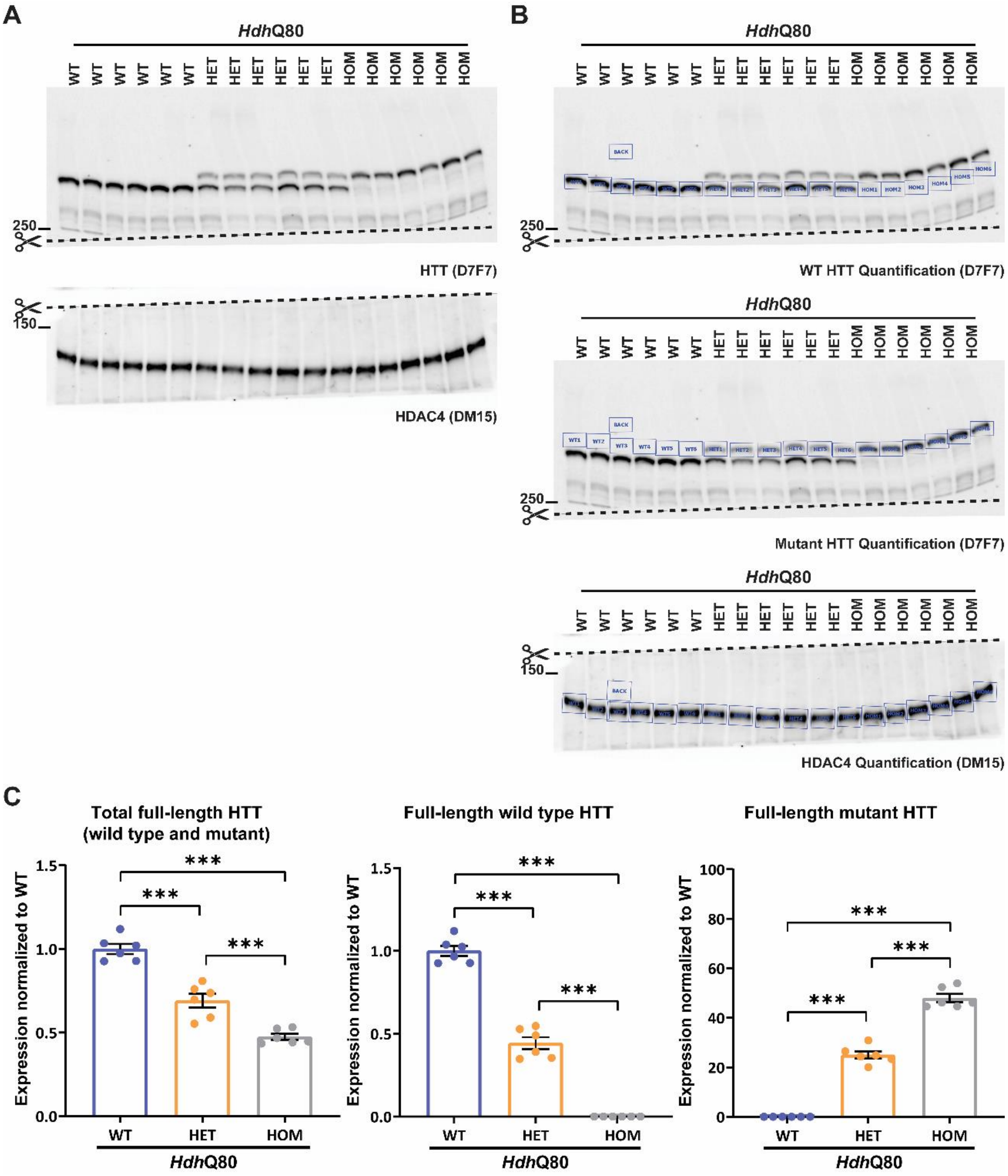

Supplementary Figure 6. Quantification of full-length HTT in cortical lysates from wild-type, heterozygous and homozygous mice from the *Hdh*Q80 colony. Uncropped blots for **Figure 1A. (A)** The western blot was cut where indicated. The top section probed with D7F7 for HTT, and the bottom section with DM15 for HDAC4 (n = 3/gender/genotype). **(B)** The signals were developed using a ChemiDoc-MP Imaging System (Bio-Rad) and quantified using Image Lab Software (Bio-Rad) as illustrated. **(C)** Total full-length HTT and full-length wild-type HTT in the heterozygous and homozygous lysates were normalized to wild-type HTT levels. Full-length mutant HTT levels in the heterozygous and homozygous lysates were normalized to wild-type background. Statistical analysis was by one-way ANOVA with Bonferroni *post hoc* correction. The test statistic, degrees of freedom and *p* values for the ANOVA are provided in **Supplementary Table 18**. Error bars are mean ± SEM. $***P \leq 0.001$. WT = wild type, HET = heterozygote, HOM = homozygote, BACK = background. Protein size markers are in kDa.

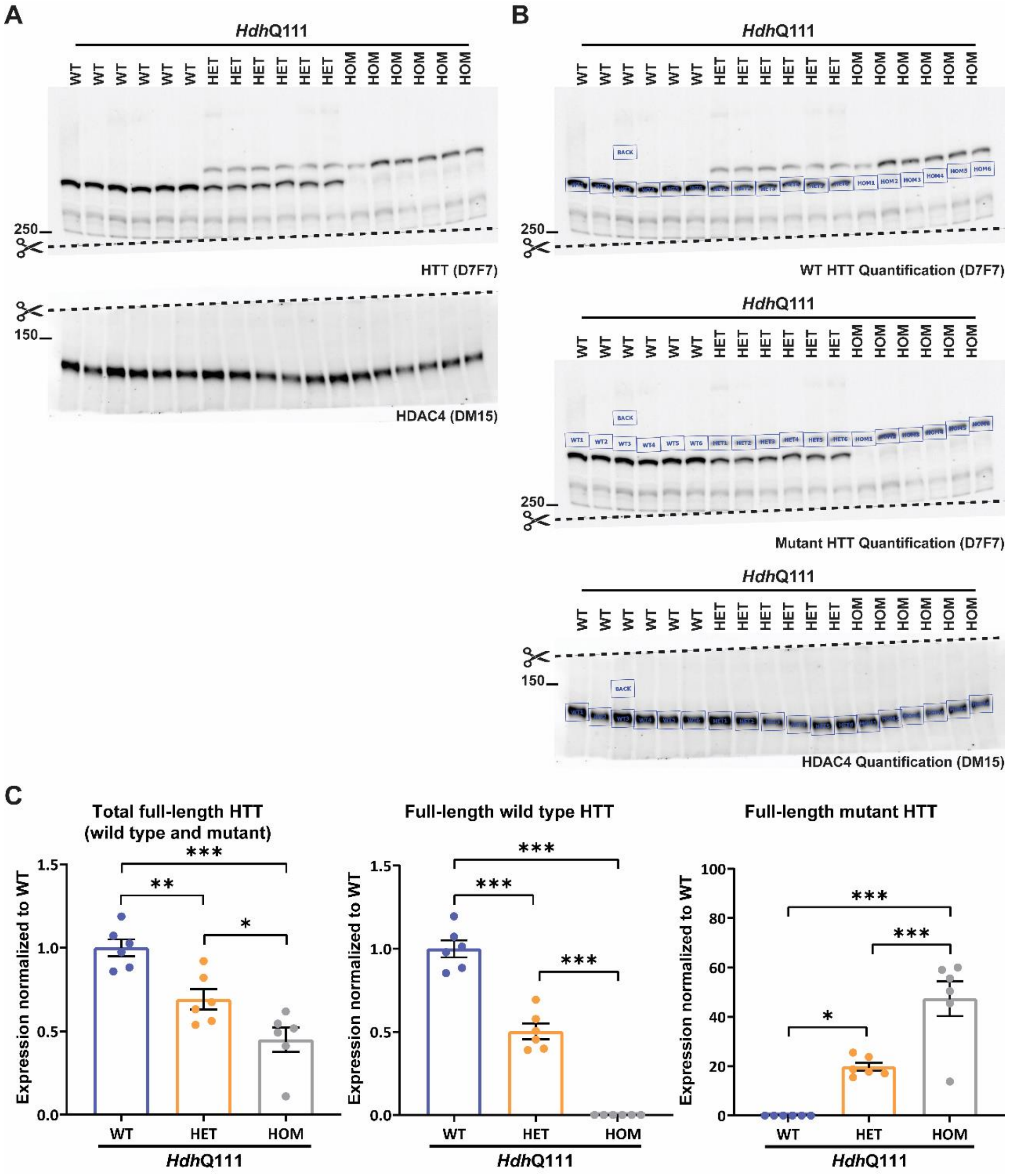

Supplementary Figure 7. Quantification of full-length HTT in cortical lysates from wild-type, heterozygous and homozygous mice from the *Hdh*Q111 colony. Uncropped blots for **Figure 1A. (A)** The western blot was cut where indicated. The top section probed with D7F7 for HTT, and the bottom section with DM15 for HDAC4 (n = 3/gender/genotype). **(B)** The signals were developed using a ChemiDoc-MP Imaging System (Bio-Rad) and quantified using Image Lab Software (Bio-Rad) as illustrated. **(C)** Total full-length HTT and full-length wild-type HTT in the heterozygous and homozygous lysates were normalized to wild-type HTT levels. Full-length mutant HTT levels in the heterozygous and homozygous lysates were normalized to wild-type background. Statistical analysis was by one-way ANOVA with Bonferroni *post hoc* correction. The test statistic, degrees of freedom and *p* values for the ANOVA are provided in **Supplementary Table 19**. Error bars are mean ± SEM. $*P \leq 0.05$, $**P \leq 0.01$, $***P \leq 0.001$. WT = wild type, HET = heterozygote, HOM = homozygote, BACK = background. Protein size markers are in kDa.

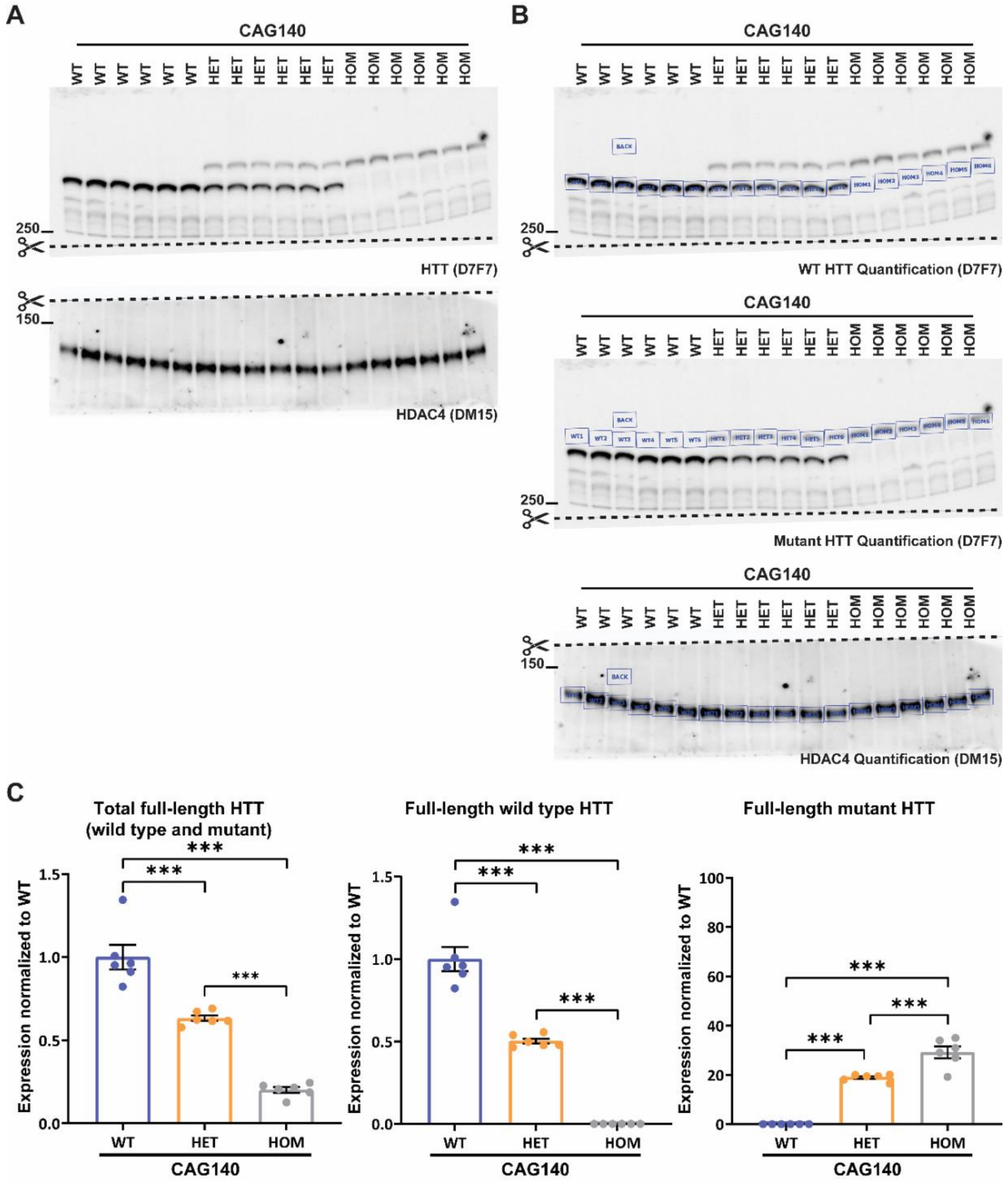

Supplementary Figure 8. Quantification of full-length HTT in cortical lysates from wild-type, heterozygous and homozygous mice from the CAG140 colony. Uncropped blots for **Figure 1A. (A)** The western blot was cut where indicated. The top section probed with D7F7 for HTT, and the bottom section with DM15 for HDAC4 (n = 3/gender/genotype). **(B)** The signals were developed using a ChemiDoc-MP Imaging System (Bio-Rad) and quantified using Image Lab Software (Bio-Rad) as illustrated. **(C)** Total full-length HTT and full-length wild-type HTT in the heterozygous and homozygous lysates were normalized to wild-type HTT levels. Full-length mutant HTT levels in the heterozygous and homozygous lysates were normalized to wild-type background. Statistical analysis was by one-way ANOVA with Bonferroni *post hoc* correction. The test statistic, degrees of freedom and *p* values for the ANOVA are provided in **Supplementary Table 20**. Error bars are mean ± SEM. ***$P \leq 0.001$. WT = wild type, HET = heterozygote, HOM = homozygote, BACK = background. Protein size markers are in kDa.

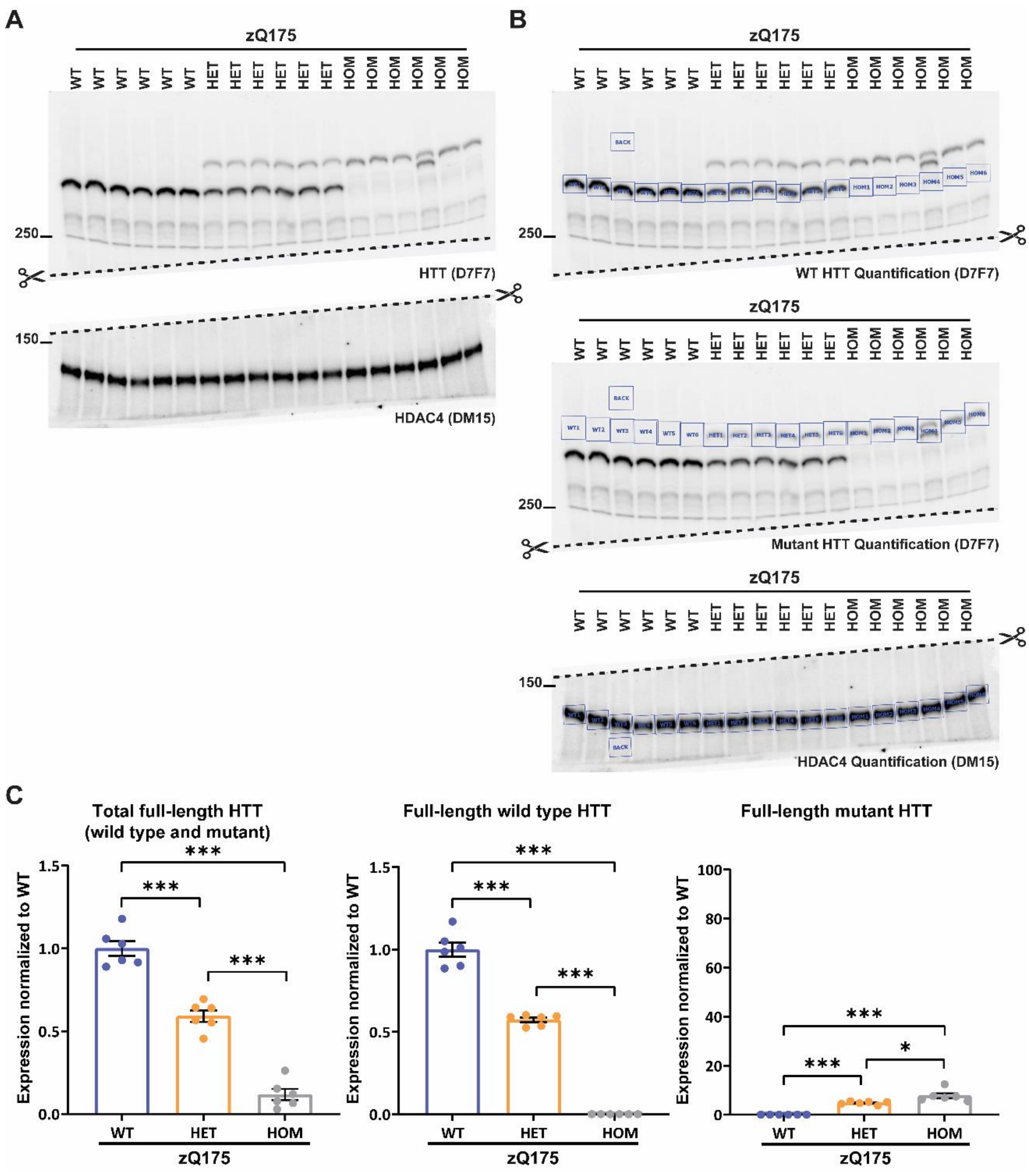

Supplementary Figure 9. Quantification of full-length HTT in cortical lysates from wild-type, heterozygous and homozygous mice from the zQ175 colony. Uncropped blots for **Figure 1A. (A)** The western blot was cut where indicated. The top section probed with D7F7 for HTT, and the bottom section with DM15 for HDAC4 (n = 3/gender/genotype). **(B)** The signals were developed using a ChemiDoc-MP Imaging System (Bio-Rad) and quantified using Image Lab Software (Bio-Rad) as illustrated. **(C)** Total full-length HTT and full-length wild-type HTT in the heterozygous and homozygous lysates were normalized to wild-type HTT levels. Full-length mutant HTT levels in the heterozygous and homozygous lysates were normalized to wild-type background. Statistical analysis was by one-way ANOVA with Bonferroni *post hoc* correction. The test statistic, degrees of freedom and *p* values for the ANOVA are provided in **Supplementary Table 21**. Error bars are mean ± SEM. *$P \leq 0.05$, ***$P \leq 0.001$. WT = wild type, HET = heterozygote, HOM = homozygote, BACK = background. Protein size markers are in kDa.

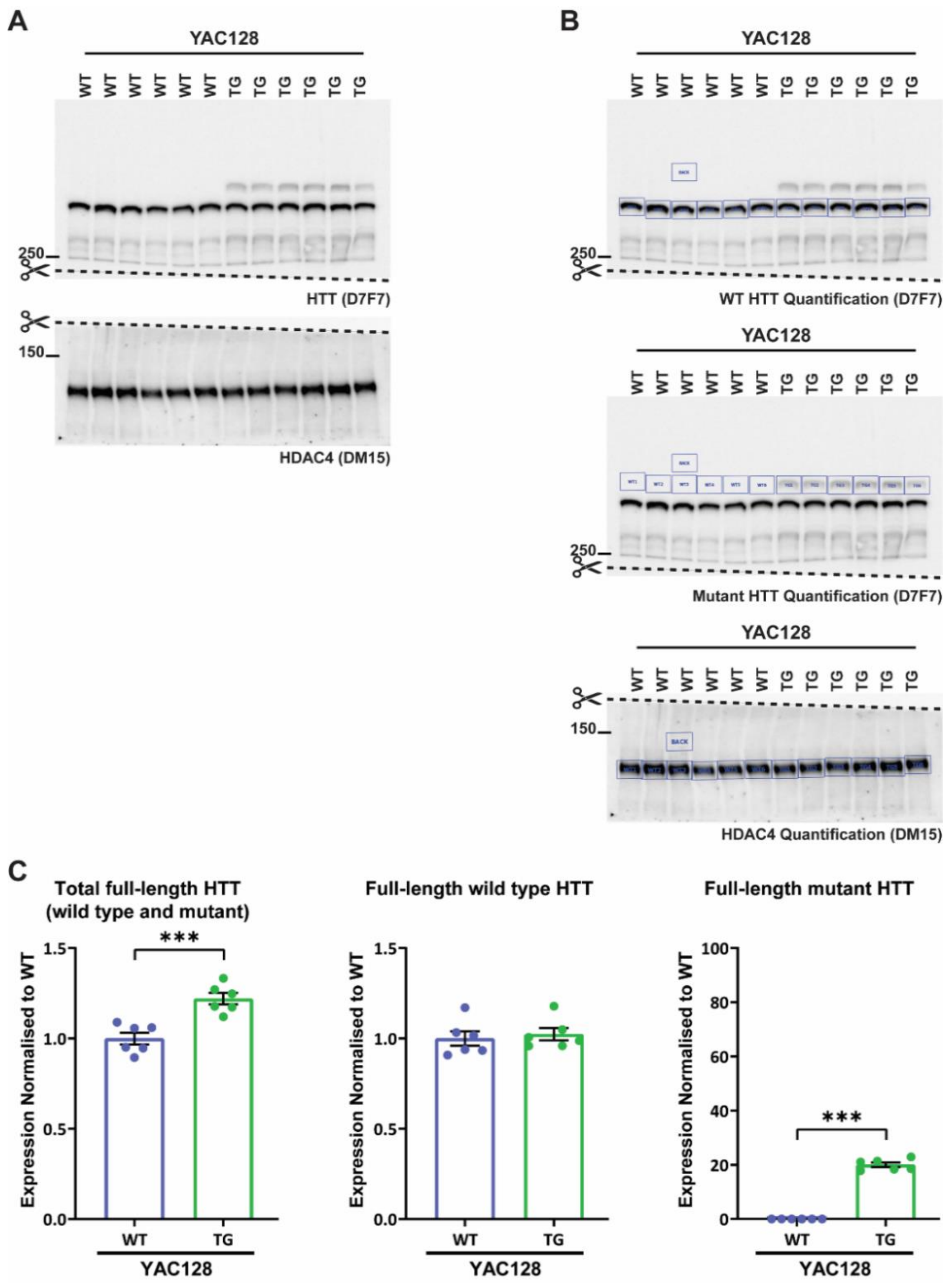

Supplementary Figure 10. Quantification of full-length HTT in cortical lysates from wild-type and transgenic mice from the YAC128 colony. Uncropped blots for **Figure 1D. (A)** The western blot was cut where indicated. The top section probed with D7F7 for HTT, and the bottom section with DM15 for HDAC4 (n = 3/gender/genotype). **(B)** The signals were developed using a ChemiDoc-MP Imaging System (Bio-Rad) and quantified using Image Lab Software (Bio-Rad) as illustrated. **(C)** Total full-length HTT and full-length wild-type HTT in the transgenic lysates were normalized to wild-type HTT levels. Full-length mutant HTT levels in the transgenic lysates were normalized to wild-type background. Statistical analysis was by Student's *t*-test. The test statistic, degrees of freedom and *p* values for the Student's *t*-tests are provided in **Supplementary Table 22**. Error bars are mean ± SEM. ****P* ≤ 0.001. WT = wild type, TG = transgenic, BACK = background. Protein size markers are in kDa.

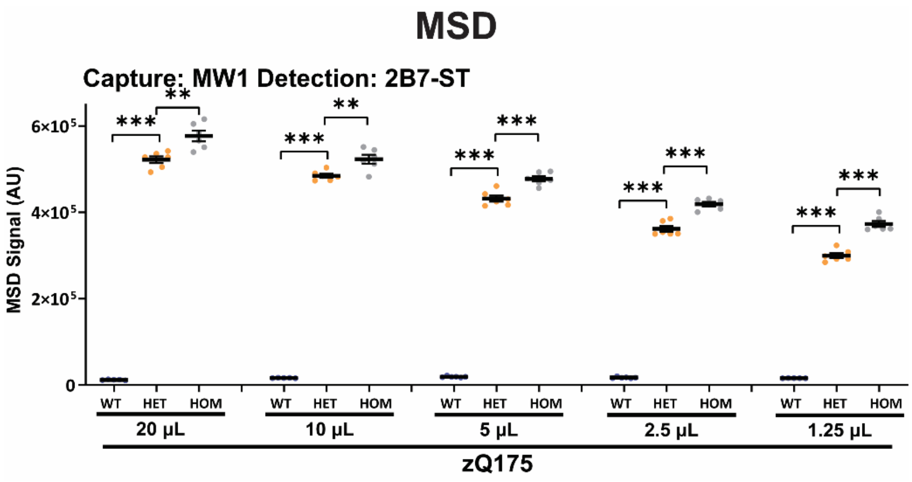

Supplementary Figure 11. The MW1-2B7 MSD signal was not saturated in lysates from heterozygous knock-in mice. The antibody pairing of MW1-2B7 was tested by MSD in decreasing serial dilutions of cortical lysates from wild-type, heterozygous and homozygous zQ175 mice at 11 weeks of age (n = 3/gender/genotype). The signal was always higher in the homozygous lysates, indicating that the heterozygous signal was not saturated. Statistical analysis was by one-way ANOVA with Bonferroni *post hoc* correction. The test statistic, degrees of freedom and *p* values for the ANOVA are provided in **Supplementary Table 23**. Error bars are mean ± SEM. ***P* ≤ 0.01, ****P* ≤ 0.001. WT = wild type, HET = heterozygote, HOM = homozygote.

Supplementary Figure 12.

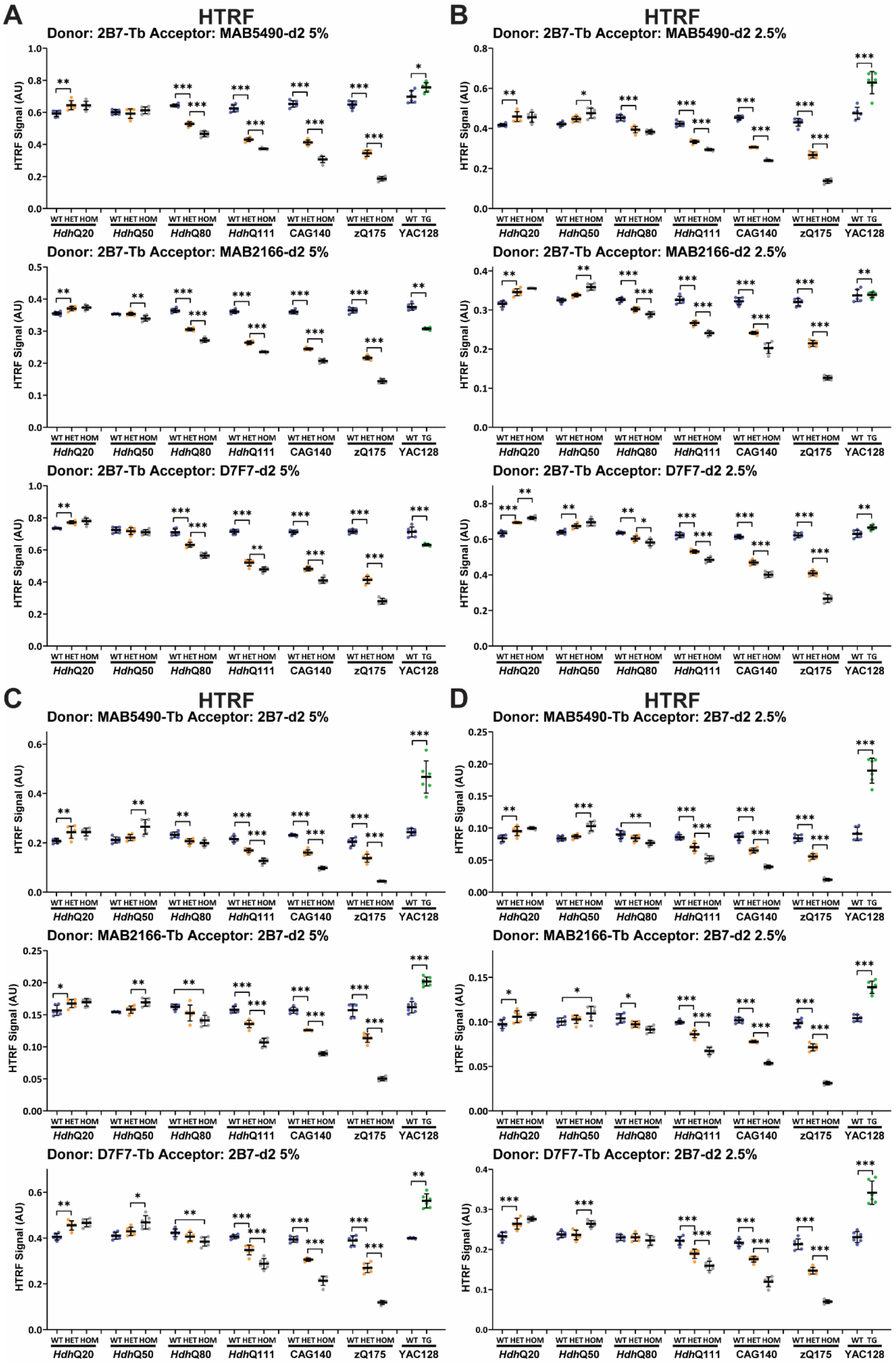

Supplementary Figure 12. The effect of lysate concentration on the HTRF assays that use 2B7 and detect total full-length HTT (mutant and wild type). Antibody pairings of 2B7-MAB5490, 2B7-MAB2166 and 2B7-D7F79 were tested by HTRF with 5% lysate **(A)** and 2.5% lysate **(B),** and of MAB5490-2B7, MAB2166-2B7 and D7F7-2B7 were tested by HTRF with 5% lysate **(C)** and 2.5% lysate **(D)** using cortical lysates from wild-type, heterozygous and homozygous *Hdh*Q20, *Hdh*Q50, *Hdh*Q80, *Hdh*Q111, CAG140 and zQ175 mice at 11 weeks of age and YAC128 and wild-type littermates at 9 weeks of age (n = 3/gender/genotype). Statistical analysis was Student's *t*-test (for YAC128) or one-way ANOVA with Bonferroni *post hoc* correction per mouse line. The test statistic, degrees of freedom and *p* values for the ANOVA are provided in **Supplementary Table 24**. Error bars are mean ± SEM. *$P \leq 0.5$, **$P \leq 0.01$, ***$P \leq 0.001$. WT = wild type, HET = heterozygote, HOM = homozygote, TG = transgenic.

Supplementary Table 6. The test statistic, degrees of freedom and *P* values for the one-way ANOVA and Student's *t*-test data presented in **Figure 1**.

	**One-Way ANOVA**												**Student's *t*-test**	
**Western Blot**	***Hdh*Q20**		***Hdh*Q50**		***Hdh*Q80**		***Hdh*Q111**		**CAG140**		**zQ175**		**YAC128**	
**Total full-length HTT expression (WT and mutant)**	$F(2, 15) = 4.511$	$P=0.0292$	$F(2, 15) = 0.3591$	$P=0.70$	$F(2, 15) = 72.90$	$P<0.001$	$F(2, 15) = 19.60$	$P<0.001$	$F(2, 15) = 80.57$	$P<0.001$	$F(2, 15) = 133.6$	$P<0.001$	$t=4.873$	$df=10$

Supplementary Table 7. The test statistic, degrees of freedom and *P* values for the one-way ANOVA and Student's *t*-test data presented in **Figure 2**.

	**One-Way ANOVA**												**Student's *t*-test**	
**qPCR**	***Hdh*Q20**		***Hdh*Q50**		***Hdh*Q80**		***Hdh*Q111**		**CAG140**		**zQ175**		**YAC128**	
Full-length *Htt* – 3' untranslated region	$F(2, 15) = 1.495$	$P=0.26$	$F(2, 15) = 1.078$	$P=0.37$	$F(2, 14) = 0.2882$	$P=0.75$	$F(2, 15) = 1.929$	$P=0.18$	$F(2, 14) = 55.44$	$P<0.001$	$F(2, 15) = 37.81$	$P<0.001$	$t=0.3584$	$df=9$
HTT1a – intron 1	$F(2, 15) = 12.92$	$P<0.001$	$F(2, 15) = 37.28$	$P<0.001$	$F(2, 15) = 175.6$	$P<0.001$	$F(2, 15) = 776.6$	$P<0.001$	$F(2, 15) = 269.1$	$P<0.001$	$F(2, 15) = 137.9$	$P<0.001$	-	
**RNA Seq**	**One-Way ANOVA (All Mouse lines as compared to WT)**													
Full-length *Htt*	$F(6, 44) = 13.29$		$P<0.001$											
HTT1a – intron 1	$F(5, 37) = 66.44$		$P<0.001$											

Supplementary Table 8. The test statistic, degrees of freedom and *P* values for the one-way ANOVA and Student's *t*-test data presented in **Figure 3**.

	**One-Way ANOVA**												**Student's *t*-test**	
**HTRF**	***Hdh*Q20**		***Hdh*Q50**		***Hdh*Q80**		***Hdh*Q111**		**CAG140**		***Hdh*Q20**		**YAC128**	
MW1-Tb: MAB5490-d2	F (2, 15) = 1.667	*p*=0.22	F (2, 15) = 422.4	*p*=<0.001	F (2, 14) = 1769	*p*=<0.001	F (2, 15) = 1129	*p*=<0.001	F (2, 14) = 1114	*p*=<0.001	F (2, 15) = 1556	*p*=<0.001	t=58.12	df=10
MW1-Tb: MAB2166-d2	F (2, 15) = 0.9333	P=0.41	F (2, 15) = 253.3	P<0.001	F (2, 15) = 977.6	P<0.001	F (2, 15) = 822.9	P<0.001	F (2, 15) = 1706	P<0.001	F (2, 15) = 2348	P<0.001	t=47.86	df=10
MW1-Tb: D7F7-d2	F (2, 15) = 0.2011	P=0.82	F (2, 14) = 516.6	P<0.001	F (2, 14) = 885.5	P<0.001	F (2, 14) = 2131	P<0.001	F (2, 15) = 566.8	P<0.001	F (2, 14) = 466.4	P<0.001	t=42.50	df=10
MAB5490-Tb: MW1-d2	F (2, 15) = 0.8962	P=0.43	F (2, 15) = 1492	P<0.001	F (2, 15) = 2456	P<0.001	F (2, 15) = 1746	P<0.001	F (2, 15) = 2683	P<0.001	F (2, 15) = 1661	P<0.001	t=84.44	df=10
MAB2166-Tb: MW1-d2	F (2, 15) = 0.8865	P=0.43	F (2, 15) = 196.5	P<0.001	F (2, 15) = 1499	P<0.001	F (2, 13) = 745.3	P<0.001	F (2, 15) = 2294	P<0.001	F (2, 15) = 1074	P<0.001	t=26.67	df=10
D7F7-Tb: MW1-d2	F (2, 13) = 13.46	P<0.001	F (2, 15) = 1389	P<0.001	F (2, 14) = 839.7	P<0.001	F (2, 15) = 2692	P<0.001	F (2, 15) = 794.1	P<0.001	F (2, 15) = 892.3	P<0.001	t=23.19	df=10
**MSD**	***Hdh*Q20**		***Hdh*Q50**		***Hdh*Q80**		***Hdh*Q111**		**CAG140**		***Hdh*Q20**		**YAC128**	
Capture: MW1 Detection: MAB5490-ST	F (2, 14) = 3.817	P=0.05	F (2, 14) = 817.7	P<0.001	F (2, 15) = 468.3	P<0.001	F (2, 15) = 374.9	P<0.001	F (2, 14) = 229.4	P<0.001	F (2, 14) = 478.9	P<0.001	t=27.02	df=10
Capture: MW1 Detection: MAB2166- ST	F (2, 15) = 2.528	P=0.11	F (2, 15) = 974.2	P<0.001	F (2, 15) = 367.7	P<0.001	F (2, 14) = 434.6	P<0.001	F (2, 15) = 136.6	P<0.001	F (2, 14) = 107.3	P<0.001	t=28.15	df=10
Capture: MW1 Detection: D7F7- ST	F (2, 15) = 40.96	P<0.001	F (2, 15) = 672.1	P<0.001	F (2, 15) = 1224	P<0.001	F (2, 15) = 733.4	P<0.001	F (2, 14) = 505.8	P<0.001	F (2, 14) = 601.3	P<0.001	t=45.51	df=9
Capture: MAB5490 Detection: MW1-ST	F (2, 14) = 5.909	P=0.01	F (2, 15) = 410.1	P<0.001	F (2, 14) = 476.6	P<0.001	F (2, 13) = 2173	P<0.001	F (2, 15) = 783.8	P<0.001	F (2, 15) = 1453	P<0.001	t=43.08	df=10
Capture: MAB2166 Detection: MW1-ST	F (2, 14) = 4.222	P=0.04	F (2, 13) = 514.6	P<0.001	F (2, 15) = 879.9	P<0.001	F (2, 14) = 1148	P<0.001	F (2, 13) = 556.4	P<0.001	F (2, 15) = 1238	P<0.001	t=42.94	df=10
Capture: D7F7 Detection: MW1-ST	F (2, 15) = 0.6251	P=0.55	F (2, 15) = 668.2	P<0.001	F (2, 15) = 1926	P<0.001	F (2, 15) = 1433	P<0.001	F (2, 13) = 1704	P<0.001	F (2, 15) = 1216	P<0.001	t=38.07	df=10

Supplementary Table 9. The test statistic, degrees of freedom and *P* values for the one-way ANOVA and Student's *t*-test data presented in **Figure 4**.

	**One-Way ANOVA**												**Student's *t*-test**	
**HTRF**	***Hdh*Q20**		***Hdh*Q50**		***Hdh*Q80**		***Hdh*Q111**		**CAG140**		**zQ175**		**YAC128**	
4C9-Tb: MAB5490-d2	F (2, 15) = 3648	P<0.001	F (2, 14) = 2613	P<0.001	F (2, 15) = 2340	P<0.001	F (2, 15) = 1955	P<0.001	F (2, 15) = 306.9	P<0.001	F (2, 15) = 587.1	P<0.001	t=75.95	df=10
4C9-Tb: MAB2166-d2	F (2, 15) = 5092	P<0.001	F (2, 15) = 2540	P<0.001	F (2, 15) = 3426	P<0.001	F (2, 15) = 1115	P<0.001	F (2, 15) = 319.6	P<0.001	F (2, 15) = 547.3	P<0.001	t=36.74	df=10
4C9-Tb: D7F7-d2	F (2, 15) = 453.9	P<0.001	F (2, 15) = 646.3	P<0.001	F (2, 13) = 710.0	P<0.001	F (2, 13) = 135.3	P<0.001	F (2, 13) = 143.4	P<0.001	F (2, 15) = 116.2	P<0.001	t=11.93	df=9
MAB5490-Tb: 4C9-488	F (2, 15) = 2506	P<0.001	F (2, 15) = 1384	P<0.001	F (2, 15) = 1795	P<0.001	F (2, 14) = 1229	P<0.001	F (2, 15) = 794.9	P<0.001	F (2, 14) = 2424	P<0.001	t=87.01	df=10
MAB2166-Tb: 4C9-488	F (2, 15) = 1944	P<0.001	F (2, 15) = 754.8	P<0.001	F (2, 15) = 751.9	P<0.001	F (2, 15) = 1355	P<0.001	F (2, 15) = 485.8	P<0.001	F (2, 15) = 550.2	P<0.001	t=15.73	df=10
D7F7-Tb: 4C9-488	F (2, 15) = 103.5	P<0.001	F (2, 14) = 1200	P<0.001	F (2, 14) = 948.5	P<0.001	F (2, 13) = 297.7	P<0.001	F (2, 13) = 1630	P<0.001	F (2, 15) = 286.3	P<0.001	t=15.31	df=10
**MSD**	***Hdh*Q20**		***Hdh*Q50**		***Hdh*Q80**		***Hdh*Q111**		**CAG140**		**zQ175**		**YAC128**	
Capture: 4C9 Detection: MAB5490-ST	F (2, 15) = 110.5	P<0.001	F (2, 15) = 261.7	P<0.001	F (2, 15) = 122.8	P<0.001	F (2, 15) = 32.20	P<0.001	F (2, 14) = 38.02	P<0.001	F (2, 15) = 81.84	P<0.001	t=13.32	df=10
Capture: 4C9 Detection: MAB2166-ST	F (2, 15) = 1069	P<0.001	F (2, 15) = 975.1	P<0.001	F (2, 15) = 546.2	P<0.001	F (2, 15) = 187.2	P<0.001	F (2, 14) = 545.6	P<0.001	F (2, 15) = 316.8	P<0.001	t=29.29	df=10
Capture: 4C9 Detection: D7F7-ST	F (2, 15) = 3040	P<0.001	F (2, 15) = 470.0	P<0.001	F (2, 15) = 788.0	P<0.001	F (2, 15) = 407.5	P<0.001	F (2, 14) = 767.8	P<0.001	F (2, 15) = 373.9	P<0.001	t=27.64	df=10
Capture: MAB5490 Detection: 4C9-ST	F (2, 14) = 614.9	P<0.001	F (2, 15) = 925.6	P<0.001	F (2, 15) = 532.8	P<0.001	F (2, 15) = 368.3	P<0.001	F (2, 15) = 234.0	P<0.001	F (2, 15) = 420.1	P<0.001	t=19.38	df=9
Capture: MAB2166 Detection: 4C9-ST	F (2, 14) = 784.0	P<0.001	F (2, 15) = 1450	P<0.001	F (2, 15) = 897.5	P<0.001	F (2, 15) = 599.4	P<0.001	F (2, 15) = 423.9	P<0.001	F (2, 15) = 1156	P<0.001	t=48.19	df=10
Capture: D7F7 Detection: 4C9-ST	F (2, 15) = 591.4	P<0.001	F (2, 15) = 649.7	P<0.001	F (2, 15) = 909.4	P<0.001	F (2, 15) = 651.3	P<0.001	F (2, 15) = 166.4	P<0.001	F (2, 15) = 73.04	P<0.001	t=24.46 ,	df=10

Supplementary Table 10. The test statistic, degrees of freedom and *P* values for the one-way ANOVA and Student's *t*-test data presented in **Figure 5**.

	**One-Way ANOVA**												**Student's *t*-test**	
**HTRF**	***Hdh*Q20**		***Hdh*Q50**		***Hdh*Q80**		***Hdh*Q111**		**CAG140**		**zQ175**		**YAC128**	
2B7-Tb: MW8-d2	F (2, 15) = 0.1677	P=0.85	F (2, 15) = 131.0	P<0.001	F (2, 15) = 581.7	P<0.001	F (2, 15) = 1073	P<0.001	F (2, 15) = 1657	P<0.001	F (2, 15) = 636.2	P<0.001	t=13.21	df=10
MW1-Tb: MW8-d2	F (2, 14) = 0.7074	P=0.51	F (2, 15) = 8.068	P=0.004	F (2, 15) = 116.6	P<0.001	F (2, 15) = 122.6	P<0.001	F (2, 15) = 1424	P<0.001	F (2, 15) = 1424	P<0.001	t=19.77	df=10

	**One-Way ANOVA**												**Student's *t*-test**					
	***Hdh*Q20**		***Hdh*Q50**		***Hdh*Q80**		***Hdh*Q111**		**CAG140**		**zQ175**		**zQ175**		**YAC128**			
	11 weeks												26 weeks		9 weeks		26 weeks	
4C9-Tb: MW8-d2	F (2, 14) = 0.5273	P=0.60	F (2, 14) = 15.48	P<0.001	F (2, 14) = 65.53	P<0.001	F (2, 15) = 312.9	P<0.001	F (2, 15) = 562.4	P<0.001	F (2, 15) = 349.0	P<0.001	t=21.74	df=10	t=25.26	df=9	t=14.00	df=9

	**One-Way ANOVA**												**Student's *t*-test**	
**MSD**	***Hdh*Q20**		***Hdh*Q50**		***Hdh*Q80**		***Hdh*Q111**		**CAG140**		**zQ175**		**YAC128**	
Capture: 2B7 Detection: MW8-ST	F (2, 15) = 0.0063	P>0.99	F (2, 15) = 2.775	P=0.09	F (2, 15) = 2.862	P=0.09	F (2, 15) = 23.79	P<0.001	F (2, 13) = 109.1	P<0.001	F (2, 14) = 426.4	P<0.001	t=1.060	df=10
Capture: MW1 Detection: MW8-ST	F (2, 15) = 0.8203	P=0.46	F (2, 15) = 41.11	P<0.001	F (2, 15) = 219.6	P<0.001	F (2, 15) = 289.4	P<0.001	F (2, 13) = 987.3	P<0.001	F (2, 14) = 477.9	P<0.001	t=17.10	df=10
Capture: 4C9 Detection: MW8-ST	F (2, 14) = 7.188	P=0.007	F (2, 14) = 0.8879	P=0.43	F (2, 15) = 0.4414	P=0.65	F (2, 15) = 76.29	P<0.001	F (2, 14) = 104.2	P<0.001	F (2, 14) = 145.9	P<0.001	t=182.2	df=9

Supplementary Table 11. The test statistic, degrees of freedom and *P* values for the one-way ANOVA and Student's *t*-test data presented in **Figure 6**.

	**One-Way ANOVA**												**Student's *t*-test**	
**HTRF**	***Hdh*Q20**		***Hdh*Q50**		***Hdh*Q80**		***Hdh*Q111**		**CAG140**		**zQ175**		**YAC128**	
2B7-Tb: MW1-d2	$F(2, 14) = 1712$	$P<0.001$	$F(2, 15) = 12651$	$P<0.001$	$F(2, 14) = 5056$	$P<0.001$	$F(2, 14) = 7272$	$P<0.001$	$F(2, 13) = 6159$	$P<0.001$	$F(2, 12) = 4103$	$P<0.001$	t=198.2	df=10
2B7-Tb: 4C9-488	$F(2, 15) = 24076$	$P<0.001$	$F(2, 15) = 8816$	$P<0.001$	$F(2, 15) = 8060$	$P<0.001$	$F(2, 14) = 2391$	$P<0.001$	$F(2, 15) = 4239$	$P<0.001$	$F(2, 15) = 6117$	$P<0.001$	t=51.31	df=10
MW1-Tb: 2B7-d2	$F(2, 15) = 7.750$	$P=0.005$	$F(2, 14) = 1684$	$P<0.001$	$F(2, 15) = 4800$	$P<0.001$	$F(2, 15) = 3370$	$P<0.001$	$F(2, 13) = 2159$	$P<0.001$	$F(2, 13) = 2841$	$P<0.001$	t=70.39	df=9
MW1-Tb: 4C9-488	$F(2, 15) = 5.421$	$P=0.02$	$F(2, 15) = 77.80$	$P<0.001$	$F(2, 15) = 613.8$	$P<0.001$	$F(2, 15) = 532.0$	$P<0.001$	$F(2, 15) = 1159$	$P<0.001$	$F(2, 15) = 686.2$	$P<0.001$	t=38.93	df=10
4C9-Tb: 2B7-d2	$F(2, 15) = 5185$	$P<0.001$	$F(2, 15) = 3340$	$P<0.001$	$F(2, 15) = 2706$	$P<0.001$	$F(2, 15) = 1394$	$P<0.001$	$F(2, 14) = 344.8$	$P<0.001$	$F(2, 15) = 415.7$	$P<0.001$	t=100.3	df=10
4C9-Tb: MW1-d2	$F(2, 15) = 2.622$	$P=0.11$	$F(2, 15) = 2359$	$P<0.001$	$F(2, 15) = 1207$	$P<0.001$	$F(2, 15) = 1534$	$P<0.001$	$F(2, 13) = 1089$	$P<0.001$	$F(2, 14) = 702.7$	$P<0.001$	t=46.96	df=10
**MSD**	***Hdh*Q20**		***Hdh*Q50**		***Hdh*Q80**		***Hdh*Q111**		**CAG140**		**zQ175**		**YAC128**	
Capture: 2B7 Detection: MW1-ST	$F(2, 15) = 4.615$	$P=0.03$	$F(2, 15) = 4.934$	$P=0.02$	$F(2, 14) = 136.2$	$P<0.001$	$F(2, 15) = 197.6$	$P<0.001$	$F(2, 13) = 74.54$	$P<0.001$	$F(2, 15) = 188.2$	$P<0.001$	t=8.491	df=10
Capture: 2B7 Detection: 4C9-ST	$F(2, 13) = 262.8$	$P<0.001$	$F(2, 15) = 535.1$	$P<0.001$	$F(2, 15) = 352.5$	$P<0.001$	$F(2, 14) = 239.8$	$P<0.001$	$F(2, 12) = 358.7$	$P<0.001$	$F(2, 12) = 1546$	$P<0.001$	t=13.84	df=9
Capture: MW1 Detection: 2B7-ST	$F(2, 15) = 11.98$	$P<0.001$	$F(2, 15) = 996.9$	$P<0.001$	$F(2, 15) = 3464$	$P<0.001$	$F(2, 14) = 1497$	$P<0.001$	$F(2, 15) = 1065$	$P<0.001$	$F(2, 15) = 772.5$	$P<0.001$	t=91.57	df=9
Capture: MW1 Detection: 4C9-ST	$F(2, 15) = 0.7315$	$P=0.50$	$F(2, 15) = 513.1$	$P<0.001$	$F(2, 15) = 520.0$	$P<0.001$	$F(2, 15) = 240.1$	$P<0.001$	$F(2, 13) = 227.4$	$P<0.001$	$F(2, 15) = 580.5$	$P<0.001$	t=40.57	df=10
Capture: 4C9 Detection: 2B7-ST	$F(2, 15) = 302.7$	$P<0.001$	$F(2, 15) = 675.7$	$P<0.001$	$F(2, 15) = 576.0$	$P<0.001$	$F(2, 15) = 67.81$	$P<0.001$	$F(2, 13) = 82.23$	$P<0.001$	$F(2, 15) = 44.33$	$P<0.001$	t=12.59	df=10
Capture: 4C9 Detection: MW1-ST	$F(2, 15) = 10.14$	$P=0.002$	$F(2, 15) = 193.4$	$P<0.001$	$F(2, 15) = 1812$	$P<0.001$	$F(2, 15) = 1266$	$P<0.001$	$F(2, 13) = 1255$	$P<0.001$	$F(2, 15) = 1324$	$P<0.001$	t=47.22	df=10

Supplementary Table 12. The test statistic, degrees of freedom and *P* values for the one-way ANOVA and Student's *t*-test data presented in **Figure 7**.

	**One-Way ANOVA**												**Student's *t*-test**	
**HTRF**	***Hdh*Q20**		***Hdh*Q50**		***Hdh*Q80**		***Hdh*Q111**		**CAG140**		**zQ175**		**YAC128**	
2B7-Tb: MAB5490-d2	F (2, 15) = 9.187	P=0.002	F (2, 15) = 247.0	P<0.001	F (2, 15) = 1080	P<0.001	F (2, 15) = 1530	P<0.001	F (2, 15) = 3043	P<0.001	F (2, 15) = 4724	P<0.001	t=14.03	df=10
2B7-Tb: MAB2166-d2	F (2, 14) = 18.13	P<0.001	F (2, 15) = 341.5	P<0.001	F (2, 15) = 1118	P<0.001	F (2, 15) = 418.4	P<0.001	F (2, 15) = 1927	P<0.001	F (2, 15) = 2135	P<0.001	t=22.61	df=10
2B7-Tb: D7F7-d2	F (2, 15) = 6.395	P=0.010	F (2, 15) = 249.1	P<0.001	F (2, 14) = 616.2	P<0.001	F (2, 15) = 306.8	P<0.001	F (2, 15) = 698.8	P<0.001	F (2, 15) = 1194	P<0.001	t=11.65	df=10
MAB5490-Tb: 2B7-d2	F (2, 15) = 1.932	P=0.18	F (2, 15) = 17.82	P<0.001	F (2, 15) = 76.09	P<0.001	F (2, 15) = 820.9	P<0.001	F (2, 14) = 1160	P<0.001	F (2, 14) = 1763	P<0.001	t=1.464	df=10
MAB2166-Tb: 2B7-d2	F (2, 15) = 2.648	P=0.10	F (2, 15) = 21.42	P<0.001	F (2, 15) = 51.66	P<0.001	F (2, 15) = 113.7	P<0.001	F (2, 15) = 660.0	P<0.001	F (2, 15) = 432.7	P<0.001	t=2.605	df=10
D7F7-Tb: 2B7-d2	F (2, 14) = 8.502	P=0.004	F (2, 14) = 296.7	P<0.001	F (2, 14) = 214.7	P<0.001	F (2, 14) = 149.5	P<0.001	F (2, 15) = 394.2	P<0.001	F (2, 15) = 751.1	P<0.001	t=5.246 030912	df=10
**MSD**	***Hdh*Q20**		***Hdh*Q50**		***Hdh*Q80**		***Hdh*Q111**		**CAG140**		**zQ175**		**YAC128**	
Capture: 2B7 Detection: MAB5490-ST	F (2, 14) = 5.947	P=0.01	F (2, 15) = 4.864	P=0.02	F (2, 15) = 9.689	P=0.002	F (2, 15) = 54.38	P<0.001	F (2, 15) = 115.7	P<0.001	F (2, 15) = 477.9	P<0.001	t=4.364	df=10
Capture: 2B7 Detection: MAB2166-ST	F (2, 14) = 3.217	P=0.07	F (2, 15) = 5.465	P=0.02	F (2, 15) = 18.16	P<0.001	F (2, 13) = 16.10	P<0.001	F (2, 14) = 59.33	P<0.001	F (2, 15) = 195.2	P<0.001	t=1.310	df=10
Capture: 2B7 Detection: D7F7-ST	F (2, 15) = 2.580	P=0.11	F (2, 15) = 6.961	P=0.007	F (2, 14) = 64.51	P<0.001	F (2, 15) = 117.5	P<0.001	F (2, 13) = 123.7	P<0.001	F (2, 15) = 291.9	P<0.001	t=2.637	df=10
Capture: MAB5490 Detection: 2B7-ST	F (2, 13) = 26.48	P<0.001	F (2, 14) = 37.54	P<0.001	F (2, 15) = 0.6456	P=0.54	F (2, 15) = 14.60	P<0.001	F (2, 15) = 148.8	P<0.001	F (2, 15) = 233.9	P<0.001	t=9.715	df=10
Capture: MAB2166 Detection: 2B7-ST	F (2, 15) = 1.670	P=0.22	F (2, 15) = 2.898	P=0.09	F (2, 15) = 6.538	P=0.009	F (2, 15) = 15.66	P<0.001	F (2, 15) = 33.59	P<0.001	F (2, 15) = 86.95	P<0.001	t=2.057	df=10
Capture: D7F7 Detection: 2B7-ST	F (2, 15) = 7.010	P=0.007	F (2, 14) = 6.461	P=0.01	F (2, 15) = 1.320	P=0.30	F (2, 15) = 9.529	P=0.002	F (2, 15) = 44.74	P<0.001	F (2, 15) = 99.64	P<0.001	t=3.011	df=10

Supplementary Table 13. The test statistic, degrees of freedom and *P* values for the one-way ANOVA and Student's *t*-test data presented in **Figure 8**.

	**One-Way ANOVA**												**Student's *t*-test**	
**HTRF**	***Hdh*Q20**		***Hdh*Q50**		***Hdh*Q80**		***Hdh*Q111**		**CAG140**		**zQ175**		**YAC128**	
MAB5490-Tb: MAB2166-d2	F (2, 15) = 10.50	P=0.001	F (2, 15) = 61.91	P<0.001	F (2, 15) = 22.19	P<0.001	F (2, 15) = 3.546	P=0.05	F (2, 15) = 13.05	P<0.001	F (2, 13) = 173.4	P<0.001	t=14.78	df=10
MAB5490-Tb: D7f7-d2	F (2, 13) = 37.73	P<0.001	F (2, 14) = 36.69	P<0.001	F (2, 15) = 14.22	P<0.001	F (2, 15) = 1.886	P=0.19	F (2, 15) = 27.06	P<0.001	F (2, 14) = 191.3	P<0.001	t=17.55	df=10
MAB2166-Tb: MAB5490-d2	F (2, 15) = 9.516	P=0.002	F (2, 15) = 168.1	P<0.001	F (2, 15) = 7.182	P=0.006	F (2, 15) = 1.745	P=0.21	F (2, 15) = 19.92	P<0.001	F (2, 14) = 63.88	P<0.001	t=9.772	df=10
MAB2166-Tb: D7f7-d2	F (2, 14) = 2.113	P=0.16	F (2, 14) = 23.42	P<0.001	F (2, 15) = 6.307	P=0.01	F (2, 15) = 0.04436	P=0.96	F (2, 15) = 21.70	P<0.001	F (2, 14) = 247.7	P<0.001	t=5.954	df=10
D7f7-Tb: MAB5490-d2	F (2, 15) = 5.781	P=0.01	F (2, 15) = 54.58	P<0.001	F (2, 15) = 2.739	P=0.10	F (2, 14) = 3.103	P=0.08	F (2, 15) = 30.74	P<0.001	F (2, 14) = 46.69	P<0.001	t=19.79	df=10
D7F7-Tb: MAB2166-d2	F (2, 15) = 2.895	P=0.09	F (2, 15) = 51.08	P<0.001	F (2, 15) = 5.022	P=0.02	F (2, 14) = 21.12	P<0.001	F (2, 15) = 38.00	P<0.001	F (2, 14) = 754.8	P<0.001	t=11.41	df=10
**MSD**	***Hdh*Q20**		***Hdh*Q50**		***Hdh*Q80**		***Hdh*Q111**		**CAG140**		**zQ175**		**YAC128**	
Capture: MAB5490 Detection: MAB2166-ST	F (2, 15) = 4.854	P=0.02	F (2, 14) = 11.67	P=0.001	F (2, 15) = 1.543	P=0.25	F (2, 14) = 5.316	P=0.02	F (2, 14) = 24.73	P<0.001	F (2, 14) = 24.73	P<0.001	t=15.31	df=10
Capture: MAB5490 Detection: D7F7-ST	F (2, 14) = 7.013	P=0.008	F (2, 13) = 62.97	P<0.001	F (2, 14) = 8.281	P=0.004	F (2, 14) = 7.435	P=0.006	F (2, 14) = 74.56	P<0.001	F (2, 14) = 97.46	P<0.001	t=10.57	df=10
Capture: MAB2166 Detection: MAB5490-ST	F (2, 15) = 3.336	P=0.06	F (2, 15) = 4.110	P=0.04	F (2, 14) = 4.913	P=0.02	F (2, 15) = 13.67	P<0.001	F (2, 15) = 12.23	P<0.001	F (2, 15) = 45.03	P<0.001	t=8.420	df=10
Capture: MAB2166 Detection: D7F7-ST	F (2, 15) = 5.289	P=0.02	F (2, 15) = 66.47	P<0.001	F (2, 15) = 9.092	P=0.003	F (2, 15) = 5.738	P=0.01	F (2, 15) = 43.38	P<0.001	F (2, 14) = 196.3	P<0.001	t=3.696	df=10
Capture: D7F7 Detection: MAB5490-ST	F (2, 13) = 13.84	P<0.001	F (2, 14) = 102.5	P<0.001	F (2, 12) = 11.59	P=0.002	F (2, 12) = 8.750	P=0.005	F (2, 13) = 5.351	P=0.02	F (2, 13) = 40.38	P<0.001	t=9.445	df=8
Capture: D7F7 Detection: MAB2166-ST	F (2, 15) = 15.32	P<0.001	F (2, 15) = 105.5	P<0.001	F (2, 15) = 13.55	P<0.001	F (2, 15) = 12.88	P<0.001	F (2, 15) = 16.10	P<0.001	F (2, 14) = 143.6	P<0.001	t=4.909	df=10

Supplementary Table 14. The test statistic, degrees of freedom and *P* values for the one-way ANOVA and Student's *t*-test data presented in **Supplementary Figure 2**.

	**One-Way ANOVA**												**Student's *t*-test**	
**Western Blot**	***Hdh*Q20**		***Hdh*Q50**		***Hdh*Q80**		***Hdh*Q111**		**CAG140**		**zQ175**		**YAC128**	
**Total full-length HTT (WT and mutant)**	F (2, 15) = 4.511	P=0.0292	F (2, 15) = 0.3591	P=0.70	F (2, 15) = 72.90	P<0.001	F (2, 15) = 19.60	P<0.001	F (2, 15) = 80.57	P<0.001	F (2, 15) = 133.6	P<0.001	t=4.873	df=10
**Full-length wild type HTT**	-		F (2, 15) = 178.4	P<0.001	F (2, 15) = 178.4	P<0.001	F (2, 15) = 153.5	P<0.001	F (2, 15) = 331.7	P<0.001	F (2, 15) = 377.2	P<0.001	t=0.4471	df=10
**Full-length mutant HTT**	-		F (2, 15) = 142.3	P<0.001	F (2, 15) = 347.8	P<0.001	F (2, 15) = 32.15	P<0.001	F (2, 15) = 112.4	P<0.001	F (2, 15) = 42.02	P<0.001	t=20.77	df=10

Supplementary Table 15. The test statistic, degrees of freedom and *P* values for the one-way ANOVA and Student's *t*-test data presented in **Supplementary Figure 3**.

	**Student's *t*-test**					
**Western Blot**	***Hdh*Q111**		**CAG140**		**zQ175**	
Total full-length HTT (WT or mutant)	**t=5.510**	**df=6**	**t=12.08**	**df=6**	**t=9.154**	**df=6**
	**One-Way ANOVA**					
Total full-length HTT (WT or mutant)	**F (2, 15) = 27.21**		**P<0.001**			

Supplementary Table 16. The test statistic, degrees of freedom and *P* values for the one-way ANOVA data presented in **Supplementary Figure 4**.

	**One-Way ANOVA**	
**Western Blot**	***Hdh*Q20**	
Total full-length HTT (WT and mutant)	**F (2, 15) = 4.511**	**P=0.0292**

Supplementary Table 17. The test statistic, degrees of freedom and *P* values for the one-way ANOVA data presented in **Supplementary Figure 5**.

	**One-Way ANOVA**	
**Western Blot**	***Hdh*Q50**	
Total full-length HTT (WT and mutant)	**F (2, 15) = 0.3591**	**P=0.70**
Full-length wild type HTT	**F (2, 15) = 178.4**	**P<0.001**
Full-length mutant HTT	**F (2, 15) = 142.3**	**P<0.001**

Supplementary Table 18. The test statistic, degrees of freedom and *P* values for the one-way ANOVA data presented in **Supplementary Figure 6**.

	**One-Way ANOVA**	
**Western Blot**	***Hdh*Q80**	
Total full-length HTT (WT and mutant)	**F (2, 15) = 72.90**	**P<0.001**
Full-length wild type HTT	**F (2, 15) = 178.4**	**P<0.001**
Full-length mutant HTT	**F (2, 15) = 347.8**	**P<0.001**

Supplementary Table 19. The test statistic, degrees of freedom and *P* values for the one-way ANOVA data presented in **Supplementary Figure 7**.

	**One-Way ANOVA**	
**Western Blot**	***Hdh*Q111**	
Total full-length HTT (WT and mutant)	**F (2, 15) = 19.60**	**P<0.001**
Full-length wild type HTT	**F (2, 15) = 153.5**	**P<0.001**
Full-length mutant HTT	**F (2, 15) = 32.15**	**P<0.001**

Supplementary Table 20. The test statistic, degrees of freedom and *P* values for the one-way ANOVA data presented in **Supplementary Figure 8**.

	**One-Way ANOVA**	
**Western Blot**	**CAG140**	
Total full-length HTT (WT and mutant)	**F (2, 15) = 80.57**	**P<0.001**
Full-length wild type HTT	**F (2, 15) = 331.7**	**P<0.001**
Full-length mutant HTT	**F (2, 15) = 112.4**	**P<0.001**

Supplementary Table 21. The test statistic, degrees of freedom and *P* values for the one-way ANOVA data presented in **Supplementary Figure 9**.

	**One-Way ANOVA**	
**Western Blot**	**zQ175**	
Total full-length HTT (WT and mutant)	**F (2, 15) = 133.6**	**P<0.001**
Full-length wild type HTT	**F (2, 15) = 377.2**	**P<0.001**
Full-length mutant HTT	**F (2, 15) = 42.02**	**P<0.001**

Supplementary Table 22. The test statistic, degrees of freedom and *P* values for the Student's *t*-test data presented in **Supplementary Figure 10**.

	**Student's *t*-test**	
**Western Blot**	**YAC128**	
Total full-length HTT (WT and mutant)	**t=4.873**	**df=10**
Full-length wild type HTT	**t=0.4471**	**df=10**
Full-length mutant HTT	**t=20.77**	**df=10**

Supplementary Table 23. The test statistic, degrees of freedom and *P* values for the one-way ANOVA data presented in **Supplementary Figure 11**.

	**One-Way ANOVA**									
	**20 µL**		**10 µL**		**5 µL**		**2.5 µL**		**1.25 µL**	
**Total full-length HTT (WT and mutant)**	**F (2, 14) = 1142**	**P<0.001**	**F (2, 14) = 1573**	**P<0.001**	**F (2, 15) = 2215**	**P<0.001**	**F (2, 15) = 1992**	**P<0.001**	**F (2, 14) = 1135**	**P<0.001**

Supplementary Table 24. The test statistic, degrees of freedom and *P* values for the one-way ANOVA and Student's *t*-test data presented in **Supplementary Figure 12**.

	**One-Way ANOVA**												**Student's *t*-test**	
**HTRF 5%**	***Hdh*Q20**		***Hdh*Q50**		***Hdh*Q80**		***Hdh*Q111**		**CAG140**		**zQ175**		**YAC128**	
2B7-Tb: MAB5490-d2	F (2, 15) = 8.826	P=0.003	F (2, 15) = 1.164	P=0.34	F (2, 15) = 257.3	P<0.001	F (2, 14) = 427.2	P<0.001	F (2, 15) = 556.2	P<0.001	F (2, 14) = 812.8	P<0.001	t=2.972	df=10
2B7-Tb: MAB2166-d2	F (2, 15) = 13.83	P<0.001	F (2, 14) = 10.65	P=0.002	F (2, 14) = 360.1	P<0.001	F (2, 15) = 1139	P<0.001	F (2, 15) = 1356	P<0.001	F (2, 14) = 1393	P<0.001	t=15.92	df=10
2B7-Tb: D7F7-d2	F (2, 14) = 17.25	P<0.001	F (2, 15) = 0.8363	P=0.45	F (2, 15) = 106.9	P<0.001	F (2, 15) = 348.3	P<0.001	F (2, 15) = 679.2	P<0.001	F (2, 14) = 856.2	P<0.001	t=5.636	df=9
MAB5490-Tb: 2B7-d2	F (2, 15) = 8.180	P=0.004	F (2, 15) = 12.32	P<0.001	F (2, 15) = 14.68	P<0.001	F (2, 15) = 108.3	P<0.001	F (2, 13) = 356.1	P<0.001	F (2, 14) = 186.1	P<0.001	t=8.185	df=10
MAB2166-Tb: 2B7-d2	F (2, 15) = 6.252	P=0.01	F (2, 14) = 13.77	P<0.001	F (2, 15) = 8.718	P=0.003	F (2, 15) = 116.7	P<0.001	F (2, 13) = 527.6	P<0.001	F (2, 14) = 291.5	P<0.001	t=8.750	df=10
D7F7-Tb: 2B7-d2	F (2, 15) = 20.93	P<0.001	F (2, 15) = 11.33	P=0.001	F (2, 15) = 6.644	P=0.009	F (2, 15) = 59.50	P<0.001	F (2, 14) = 230.4	P<0.001	F (2, 14) = 306.7	P<0.001	t=11.67	df=9
**HTRF 2.5%**	***Hdh*Q20**		***Hdh*Q50**		***Hdh*Q80**		***Hdh*Q111**		**CAG140**		**zQ175**		**YAC128**	
2B7-Tb: MAB5490-d2	F (2, 15) = 7.531	P=0.005	F (2, 15) = 14.64	P<0.001	F (2, 15) = 45.89	P<0.001	F (2, 14) = 264.0	P<0.001	F (2, 13) = 1131	P<0.001	F (2, 14) = 462.0	P<0.001	t=5.967	df=10
2B7-Tb: MAB2166-d2	F (2, 14) = 48.52	P<0.001	F (2, 15) = 45.18	P<0.001	F (2, 15) = 64.48	P<0.001	F (2, 15) = 238.1	P<0.001	F (2, 15) = 232.5	P<0.001	F (2, 14) = 856.2	P<0.001	t=0.247	df=10
2B7-Tb: D7F7-d2	F (2, 14) = 123.7	P<0.001	F (2, 15) = 20.71	P<0.001	F (2, 15) = 24.85	P<0.001	F (2, 14) = 178.1	P<0.001	F (2, 15) = 453.3	P<0.001	F (2, 14) = 628.0	P<0.001	t=3.674	df=10
MAB5490-Tb: 2B7-d2	F (2, 14) = 12.82	P<0.001	F (2, 15) = 24.88	P<0.001	F (2, 15) = 10.01	P=0.002	F (2, 15) = 69.66	P<0.001	F (2, 13) = 159.3	P<0.001	F (2, 14) = 338.9	P<0.001	t=10.98	df=10
MAB2166-Tb: 2B7-d2	F (2, 15) = 6.576	P=0.009	F (2, 15) = 3.828	P=0.05	F (2, 15) = 13.87	P<0.001	F (2, 14) = 123.1	P<0.001	F (2, 13) = 627.3	P<0.001	F (2, 14) = 484.3	P<0.001	t=10.82	df=10
D7F7-Tb: 2B7-d2	F (2, 15) = 28.98	P<0.001	F (2, 15) = 16.28	P<0.001	F (2, 15) = 1.096	P=0.36	F (2, 15) = 47.80	P<0.001	F (2, 15) = 151.2	P<0.001	F (2, 14) = 301.5	P<0.001	t=8.460	df=10
